# Maternal serum C-reactive protein (CRP) and offspring attention deficit hyperactivity disorder (ADHD)

**DOI:** 10.1007/s00787-019-01372-y

**Published:** 2019-07-16

**Authors:** Roshan Chudal, Alan S. Brown, David Gyllenberg, Susanna Hinkka-Yli-Salomäki, Minna Sucksdorff, Heljä-Marja Surcel, Subina Upadhyaya, Andre Sourander

**Affiliations:** 1 Department of Child Psychiatry, Research Centre for Child Psychiatry, Institute of Clinical Medicine, Faculty of Medicine, University of Turku, Lemminkäisenkatu 3/Teutori (3rd Floor), 20014 Turku, Finland; 2 Department of Psychiatry, New York State Psychiatric Institute, Columbia University Medical Center, New York, NY USA; 3 Department of Epidemiology, Mailman School of Public Health, Columbia University, New York, NY USA; 4 Department of Adolescent Psychiatry, Helsinki University Central Hospital, Helsinki, Finland; 5 National Institutes of Health and Welfare, Helsinki, Finland; 6 Faculty of Medicine, Medical Research Center, University of Oulu, Oulu, Finland; 7 Biobank Borealis of Northern Finland, Oulu University Hospital, Oulu, Finland; 8 Department of Child Psychiatry, Turku University Hospital, Turku, Finland

**Keywords:** CRP, ADHD, Epidemiology, Prenatal inflammation, Nationwide cohort

## Abstract

Exposure to infection and inflammation during the fetal period are associated with offspring neuropsychiatric disorders. Few previous studies have examined this association with ADHD with mixed findings. This study aims to examine the association between early gestational maternal C-reactive protein (CRP), prospectively assayed in stored maternal sera and the risk of ADHD in offspring. This study is based on the Finnish Prenatal studies of ADHD (FIPS-ADHD) with a nested case–control design. It includes all singleton-born children in Finland between January 1, 1998 and December 31, 1999 and diagnosed with ADHD. A total of 1079 cases and equal number of controls were matched on date of birth, sex and place of birth. Maternal CRP levels were assessed using a latex immunoassay from archived maternal serum specimens, collected during the first and early second trimester of pregnancy. Elevated maternal CRP when analyzed as a continuous variable was not associated with offspring ADHD (OR 1.05, 95% CI 0.96–1.15). No significant associations were seen in the highest quintile of CRP (OR 1.18, 95% CI 0.88–1.58). The results were similar in both sexes as well as among ADHD cases with or without comorbid ASD or conduct disorder. In this first study examining *CRP*,* a biomarker for inflammation*, during early pregnancy in relation to offspring ADHD, we report no significant associations. The lack of any association, when considered with positive findings seen in ASD and schizophrenia, and negative findings in bipolar disorder suggests different pathways linking maternal immune activation and development of various neuropsychiatric disorders.

## Background

Attention deficit hyperactivity disorder (ADHD) is defined as impairment due to behavioral symptoms of inattention, hyperactivity and impulsivity and has an estimated worldwide prevalence of around 5% [1]. Despite having a strong genetic component, several environmental factors contribute to the development of ADHD [2].

An exaggerated central nervous system inflammatory response to a pre-/perinatal insult in the developing fetus has been hypothesized as a potential cause of ADHD [3]. Studies showing an association between maternal diseases with an immune component and offspring ADHD provide support to the role of maternal immune activation during pregnancy in development of ADHD [4–6]. In a Norwegian nationwide case–control study, maternal diagnoses of multiple sclerosis, rheumatoid arthritis, asthma, hypothyroidism, and type 1 diabetes were associated with an increased risk of ADHD in offspring [4]. Diabetes mellitus among mothers [6] as well as in both parents [5] was associated with offspring ADHD.

Exposure to infection and inflammation during the fetal period is associated with offspring schizophrenia [7], bipolar disorder (BPD) [8] and autism spectrum disorder (ASD) [9, 10] in both population-based epidemiological studies [8, 9, 11] as well as biomarker studies [7, 10]. Few previous studies have examined this association with ADHD with mixed findings. In a Colombian clinical study of 200 ADHD children using a retrospective survey, severe respiratory viral infection during pregnancy was associated with offspring ADHD [12]. In an Italian retrospective survey among 71 ADHD cases, the frequency of viral rash during pregnancy was reported significantly higher (*p* < 0.01) by mothers of ADHD cases than controls [13]. A birth cohort study in the US with 7911 ADHD cases demonstrated an association between maternal genitourinary (GU) infection and ADHD [14]. An Australian population-based case–control study of 12,991 ADHD cases showed an increased odds of offspring ADHD with maternal urinary tract infection (UTI) during pregnancy [15]. A Danish nationwide cohort study on 90,000 pregnancies showed no overall association with fever or infections during pregnancy. However, when stratifying by gestational periods, fever during early gestation, i.e., weeks 9–12 and genitourinary infections in late gestation (33–36 weeks) were associated with increased ADHD rates [16]. A recent Swedish nationwide cohort study showed that maternal infection during pregnancy requiring hospitalization was associated with ADHD in offspring. However, the finding was fully attenuated when assessed using sibling comparison [17]. A Danish twin study of 166 discordant ADHD pairs using fetal blood sample obtained 5 days after birth, showed transplacental acquired pneumococcal antibodies to be more common among higher scoring ADHD twin as compared to lower scoring twin when assessed with the Child Behavior Checklist (CBCL) and Strengths and Difficulties Questionnaire (SDQ) (*p* = 0.004) [18].

The clinical studies [12, 13] with a retrospective design are likely to be influenced by recall bias. Other larger studies have focused on the association specifically only with genitourinary infections and did not adjust for confounding due to parental psychopathology [14, 15]. The two previous nationwide studies [16, 17] did not report any association between fever or infection during pregnancy (IDP) and ADHD. Information on fever and maternal infections in the Danish study [16] was based on maternal self-report which is prone to recall bias. Ginsberg et al. [17] defined maternal IDP as infections during pregnancy requiring hospitalization identified from the nationwide registers and is thus likely that less severe infections that were not treated or treated in outpatient services were missed. They [17] also examined a range of infection categories and not a general immune activation measure (e.g., CRP, cytokines), which is more likely to represent the underlying pathway to development of neuropsychiatric disorders [19]. In addition, these studies [16, 17] used clinical reports of infection and did not have any documented biological marker of maternal infection. The only previous study using any biomarker, i.e., pneumococcal antibodies [18] used fetal samples obtained 5 days after birth and not maternal biomarkers of antenatal influences.

C-reactive protein (CRP), primarily synthesized by hepatocytes, is a well-established and reliable general marker of inflammation from both infectious and noninfectious exposures [20]. The aim of this study is to examine the association between early gestational maternal CRP, prospectively assayed in stored maternal sera and the risk of ADHD in offspring. Based on the findings from previous nationwide samples [16, 17], we hypothesize that elevated CRP levels will not be associated with offspring ADHD.

## Methods

This study is based on the Finnish Prenatal studies of ADHD (FIPS-ADHD) with a nested case–control design. The study includes all singleton-born children in Finland between January 1, 1998 and December 31, 1999 and diagnosed with ADHD, as identified from the Finnish Hospital Discharge Register (FHDR) by December 31st, 2011.

### Finnish maternity cohort

All offsprings in the FIPS-ADHD were derived from the Finnish Maternity Cohort (FMC). The FMC consists of around 2 million maternal serum samples collected during the first and early second trimester of pregnancy (5th–95th percentile: months 2–4 of pregnancy) from over 950,000 women covering virtually all pregnancies in Finland. Following informed consent, blood samples were collected at the Finnish maternity clinics to screen for congenital infections (HIV, hepatitis B, and syphilis). The median gestational age of serum collection in this study was 10 weeks (interquartile range 8–12 weeks). After the screening, approximately 1–3 mL of serum from each pregnancy are stored at − 25 °C in a protected biorepository at Biobank Borealis in Oulu, Finland, and can be used for scientific research [21]. The FMC can be linked with other Finnish nationwide registers using a unique personal identification code (PIC), which has been assigned to all residents of Finland since 1971.

### Finnish nationwide registers

The Population Information System established by the Finnish Population Register Center (PRC) is a computerized national register containing basic information about Finnish citizens and foreign permanent residents. The personal data included in the system include: name, PIC, address, citizenship and native language, family relations and date of birth and death (if applicable). The Finnish Medical Birth Register (FMBR) includes comprehensive and standardized data on the perinatal period for all live births, and stillbirths of fetuses with birth weight of at least 500 g or gestational age of at least 22 weeks. Statistics Finland is the public authority established particularly for statistical services in Finland. The FHDR contains computerized data on recorded inpatient diagnoses in Finland since 1967 and since 1998, the FHDR also includes outpatient care in public specialized hospital units. The diagnostic classification in Finland is based on the International Classification of Diseases (ICD); the 10th Revision has been used since 1996 [22]. From 1987 to 1995 the diagnoses were coded according to ICD-9 [23] and from 1969 to 1986 according to ICD-8 [24]. A previous validation study has shown the FHDR diagnosis to have 88% validity for ADHD diagnosis [25].

### Cases’ and controls’ identification

The Finnish public health care system covers both primary health care and specialized health services with children’s mental health care services provided free of charge. ADHD is typically diagnosed based on the assessment of a specialist in psychiatry or neurology in public outpatient services.

ADHD cases included in the study were singletons born in Finland between 1998 and 1999 and registered in the FHDR with the ICD-10 codes of hyperkinetic disorders F90.0, F90.1, F90.8, and F90.9 until 2011. Controls were singletons born in Finland without a diagnosis of ADHD, conduct disorder, or severe or profound intellectual disability in the FHDR and matched to the case on date of birth (± 30 days), sex, and place of birth. The controls were required to be alive and living in Finland at the date of diagnosis of the matched case. Since the FMBR was established in 1987, cases and controls were selected from those born 1998–1999 to ensure greater uniformity of the serum specimens with regard to the time of storage. Out of the 1320 total cases and controls identified, sufficient serum was available in the FMC for 1079 cases and matched controls (*n* = 1079).

### CRP assay

CRP was measured, blind to case/control status, on the clinical chemistry analyzer Architect c8200 (Abbott Laboratories, Abbott Park, IL, USA) using a latex immunoassay (Sentinel, Milan, Italy). *The CRP assays were performed 18 years after sampling*. The precision between series expressed as the coefficient of variation (mean ± SD) was 5.1% ± 2.3% and the systematic error (bias) (mean ± SD) was 2.7% ± 7.4 during the course of the study. Assay sensitivity is 0.10 mg/L.

### Covariates

Potential confounders that have been shown to be associated with both CRP levels and ADHD were initially selected. They included: maternal age [26, 27], paternal age [26, 27], maternal substance abuse diagnosis [28, 29], maternal and paternal psychopathology (excluding ADHD and substance use) [29, 30], gestational week of blood draw [31, 32], previous births [33, 34], gestational age [31, 32], and maternal SES [25, 35]. There have also been studies showing associations between ADHD diagnosis and markers of inflammation but none showing associations with CRP [29, 36]. However, since there is a lack of causal association between gestational age at birth and ADHD and it could well be an intermediate factor on the pathway between prenatal inflammation and ADHD [37], it was excluded. The FMBR was used for information on number of previous births, maternal SES and maternal age. Paternal age was obtained from the PRC and gestational week of blood-draw from the FMC. Maternal and paternal psychiatric diagnoses were obtained from the FHDR. Detailed classification of covariates is shown in Table 1. The covariates were subsequently selected for inclusion in the models based on associations with both maternal CRP and ADHD at *p* ≤ 0.1, in accord with standard texts [38].” The availability of insufficient evidence at present in the literature supporting biological relationships between risk factors and ADHD limited us in using causal knowledge as a criterion for selecting confounders [39].

**Table 1:** Relationship between covariates and maternal C-reactive protein levels (≥ /< median) in control subjects

Covariates	CRP		CRP			*p* value
	≥ median		< median			
	Mean	SD	Mean	SD	*t*	
Maternal age (years)	29.92	5.17	29.21	5.45	− 2.19	**0.03**
Paternal age (years)	32.29	6.05	31.47	6.25	− 2.20	**0.03**
Gestational week of blood-draw	11.35	3.21	9.95	2.80	− 7.60	**< 0.0001**
	*N*	%	*N*	%	*χ*^2^	
Previous births					21.42	**< 0.0001**
0	174	32.16	247	45.91		
≥ 1	367	67.84	291	54.09		
History of maternal ADHD diagnosis^a^					0.99	1.0
No	540	99.82	538	100		
Yes	1	0.18	0	0		
History of maternal substance abuse^b^					0.07	0.79
No	534	98.71	530	98.51		
Yes	7	1.29	8	1.49		
History of maternal psychopathology^c^					10.46	**0.001**
No	494	91.31	457	84.94		
Yes	47	8.69	81	15.06		
History of paternal ADHD diagnosis^a,e^					1.99	0.25
No	535	99.63	535	100		
Yes	2	0.37	0	0		
History of paternal psychopathology^d,e^					0.08	0.77
No	468	87.15	463	86.54		
Yes	69	12.85	72	13.46		
Maternal SES					3.39	0.49
Upper white collar	72	13.31	73	13.57		
Lower white collar	242	44.93	228	42.38		
Blue collar	101	18.67	92	17.10		
Others	83	15.34	86	15.99		
Missing	43	7.95	59	10.97		

Statistically significant *p* values are in bold (*p* < 0.1 )

*CRP* C-reactive protein, *SD* standard deviation, *tt* test value, *χ*^*2*^ Pearson’s Chi-squared test value

^a^ICD-10: F90.X or ICD-9: 314.X

^b^ICD-8 (291, 303, 304), ICD-9 (291, 292, 303, 304, 305) or ICD-10 (F10–19)

^c^ICD-8 (291–308), ICD-9 (291–316) or ICD-10 (F10–99), excluding mental retardation (F70–79), excluding maternal substance abuse diagnosis

^d^ICD-8 (291–308), ICD-9 (291–316) or ICD-10 (F10–99), excluding mental retardation (F70–79)

^e^Data missing for seven controls

### Statistical analysis

The analysis of the relationship between maternal CRP and offspring ADHD was based on a nested case–control design in which the controls for each case were selected from the population at risk and matched to cases on selected factors, as described in “Case and control identification”. CRP was initially examined as a continuous measure. Due to its skewed distribution, the variable was log-transformed before analysis. To further facilitate data interpretation, we examined maternal CRP categorized into quintiles. The quintiles for the case and control groups in the analyses were derived from the cut-points of maternal CRP levels that defined the quintiles in the control group with the lowest quintile defined as the reference group. With the number of cases and controls (*n* = 1079 per group), a mean value of log-transformed CRP = 1.087 in controls, a standard deviation of 0.5 and alpha set at 5% for two-sided *t* tests, we had 80% power to detect a mean difference of 0.060 between log-transformed CRP in cases versus controls. That is to say, we had power to detect a mean value of 1.147 in cases (1.087 + 0.060) corresponding to a 6% higher value than in controls. This indicates that we had power to detect even smaller differences than the positive findings of elevated CRP noted in autism [10] and schizophrenia [7].

Since the prevalence of ADHD is much more common among males, additional analysis was conducted examining CRP as a continuous measure separately for males and females. In addition, to address any possible influences of comorbid diagnosis among ADHD cases, stratified analyses were conducted for ADHD with and without autism spectrum disorder (ASD), ADHD with and without conduct disorder (CD) and ADHD without ASD or CD.

Appropriate to the nested case–control study design, point, and interval estimates of odds ratios were obtained by fitting conditional logistic regression models for matched pairs. Statistical significance was based on *p* < 0.05. All the statistical analyses were performed with SAS software (SAS 9.4, SAS Institute, Cary, N.C.).

## Results

The study included 1079 ADHD case–control pairs with the mean age of diagnosis for cases of 7.3 years (SD 1.9, range 2–14 years). As shown in Table 1, maternal age, paternal age, gestational week of blood-draw, previous births and history of maternal psychopathology were associated with maternal CRP levels among controls. As shown in Table 2, maternal age, paternal age, previous births, history of maternal ADHD, maternal substance abuse, maternal psychopathology, maternal SES, paternal ADHD, paternal psychopathology and gestational age were associated with offspring ADHD. Therefore, in the multivariate analysis, adjustments were made only for history of maternal psychopathology, previous births, maternal age and paternal age.

**Table 2:** Relationship between covariates and ADHD in case and control subjects

Covariates	Cases		Controls			*p* value
	Mean	SD	Mean	SD	*t*	
Maternal age (years)	27.88	5.86	29.56	5.32	6.96	< **0.0001**
Paternal age (years)	30.75	6.83	31.88	6.16	1.23	< **0.0001**
Gestational week of blood-draw	10.71	3.72	10.65	3.09	−0.42	0.67
	*N*	%	*N*	%	*χ*^2^	
Previous births					15.97	< **0.0001**
0	513	47.54	421	39.02		
≥ 1	566	52.46	658	60.98		
History of maternal ADHD diagnosis^a^					4.51	**0.03**
No	1072	99.35	1078	99.91		
Yes	7	0.65	1	0.09		
History of maternal substance abuse^b^					25.34	< **0.0001**
No	1022	94.72	1064	98.61		
Yes	57	5.28	15	1.39		
History of maternal psychopathology^c^					68.26	< **0.0001**
No	801	74.24	951	88.14		
Yes	278	25.76	128	11.86		
History of paternal ADHD diagnosis^a,e^					4.61	**0.03**
No	1042	99.14	1070	99.81		
Yes	9	0.86	2	0.19		
History of paternal psychopathology^d,e^					45.15	< **0.0001**
No	793	75.45	931	86.85		
Yes	258	24.55	141	13.15		
Maternal SES					38.06	< **0.0001**
Upper white collar	73	6.77	145	13.44		
Lower white collar	431	39.94	470	43.56		
Blue collar	225	20.85	193	17.89		
Others	214	19.83	169	15.66		
Missing	136	12.60	102	9.45		

Statistically significant *p* values are in bold (*p* < 0.1 )

*SD* standard deviation, *tt* test value, *χ*^*2*^ Pearson’s Chi-squared test value

^a^ICD-10: F90.X or ICD-9: 314.X

^b^ICD-8 (291, 303, 304), ICD-9 (291, 292, 303, 304, 305) or ICD-10 (F10–19)

^c^ICD-8 (291–308), ICD-9 (291–316) or ICD-10 (F10–99), excluding mental retardation (F70–79), excluding maternal substance abuse diagnosis

^d^ICD-8 (291–308), ICD-9 (291–316) or ICD-10 (F10–99), excluding mental retardation (F70–79)

^e^Data missing for 28 cases and 7 controls

**Fig. 1:**
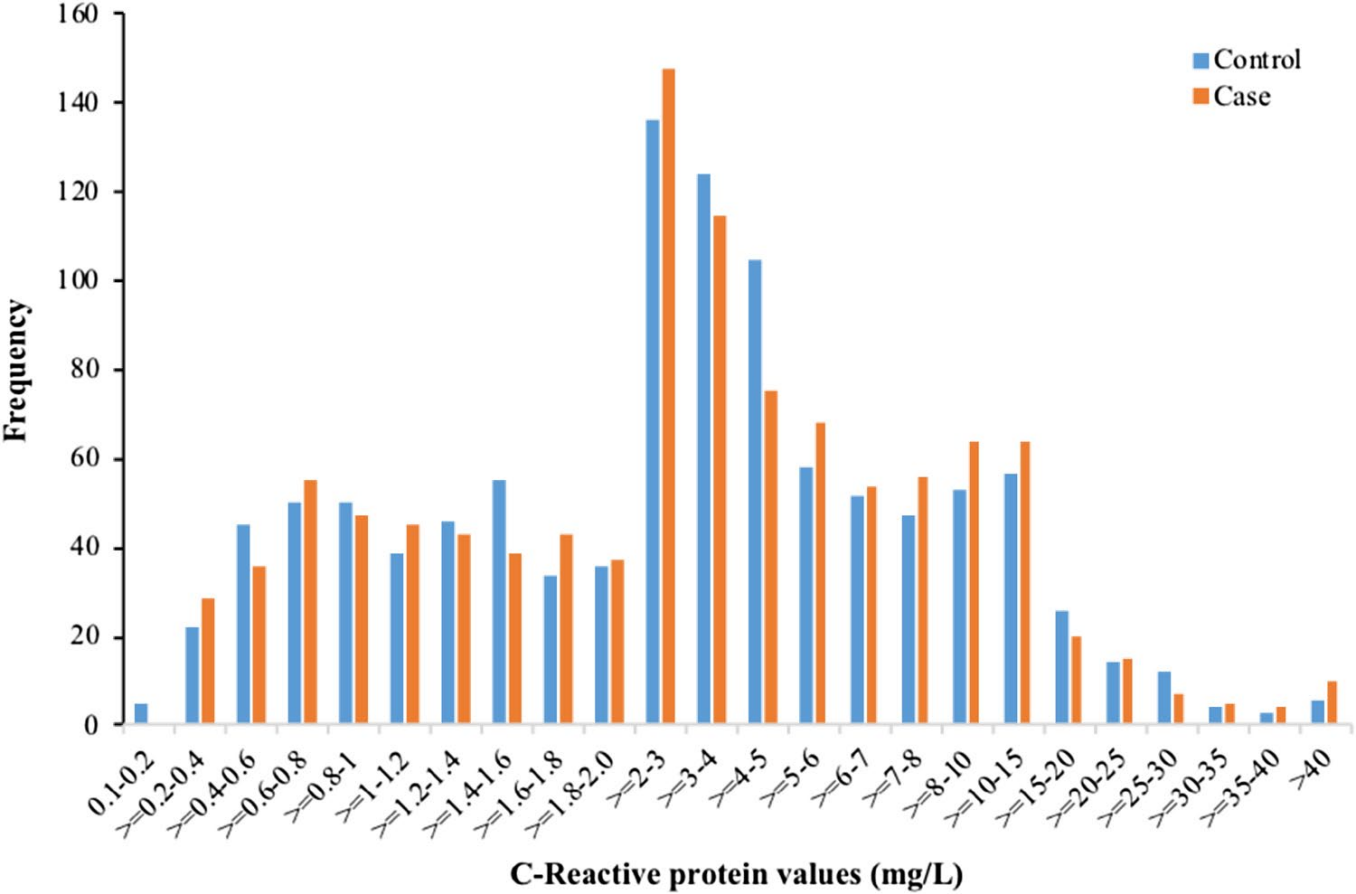
Distribution of maternal C-reactive protein levels in case and control subjects

The distribution of CRP levels among cases and controls in the sample is shown in Fig. 1. As shown in Table 3, CRP levels, when used as a continuous variable were not associated with offspring ADHD in both the unadjusted and adjusted analyses (OR 1.05, 95% CI 0.96–1.15). Furthermore, when adjustment was made only for maternal factors (psychopathology, previous births, and maternal age) the findings remained similar (OR 1.06, 95% CI 0.97–1.15). Table 4 shows the association between quintile distribution of maternal CRP and offspring ADHD. In the adjusted analysis, there was no significant association between the highest quintile (OR 1.18, 95% CI 0.88–1.58) of CRP and ADHD.

**Table 3:** Odds ratios and 95% CI of the association between maternal serum CRP (continuous, quintiles) and offspring ADHD

A. CRP as a continuous variable				
	Case (*N* = 1079)	Control (*N* = 1079)	Association with maternal serum CRP	
Maternal CRP levels (mg/L)	Median	Median	Odds ratio (OR) 95% CI	*p* value
Log-transformed analysis	3.07	3.16		
Unadjusted			1.03 (0.95–1.11)	0.47
Adjusted^a^			1.05 (0.96–1.15)	0.22

B. CRP as a categorical variable						
	Case (*N* = 1079)	Control (*N* = 1079)	Association with maternal serum CRP			
Maternal CRP levels (%) (mg/L)			Odds ratio (unadjusted) 95% CI	*p* value	Odds ratio (adjusted^a^) 95% CI	*p* value
Quintiles^b^						
< 20 (< 1.20)	212 (19.64)	211 (19.55)	Reference		Reference	
20–39 (1.20–2.38)	223 (20.66)	220 (20.39)	1.01 (0.77–1.32)	0.94	1.05 (0.791.41)	0.71
40–59 (2.39–4.02)	204 (18.90)	216 (20.01)	0.95 (0.72–1.24)	0.71	1.00 (0.75–1.35)	0.96
60–79 (4.03–7.09)	200 (18.53)	215 (19.92)	0.93 (0.71–1.22)	0.60	1.06 (0.79–1.43)	0.67
≥ 80 (≥ 7.10)	240 (22.24)	217 (20.11)	1.10 (0.84–1.44)	0.48	1.18 (0.88–1.58)	0.27

^a^Adjusted for maternal psychopathology, previous births, maternal age, and paternal age

^b^CRP values among controls in each quintile

Further analysis conducted separately for 922 males and 157 females yielded no association in both the unadjusted and adjusted analyses (adjusted OR_males_ 1.04, 95% CI 0.95–1.14; OR_females_ 1.21, 95% CI 0.93–1.57). There were 70 ADHD cases with comorbid ASD and 211 cases with comorbid conduct disorder (CD). Further analysis was conducted stratifying the sample into ADHD with ASD (OR_adj_ 1.51, 95% CI 0.97–2.36), ADHD without ASD (OR_adj_ 1.04, 95% CI 0.95–1.14), ADHD with CD (OR_adj_ 1.14, 95% CI 0.89–1.46) and ADHD without CD (OR_adj_ 1.03, 95% CI 0.94–1.14) and ADHD without ASD/CD (OR_adj_ 1.02, 95% CI 0.92–1.12).

## Discussion

This is the first study examining CRP as a biomarker for maternal inflammation during pregnancy in relation to development of ADHD in offspring. There was no association seen between maternal CRP levels and offspring ADHD.

While caution must be exercised regarding making direct comparisons between the present study and previous studies of maternal infection during pregnancy (IDP), our study findings are in line with two previous nationwide studies that did not report any association overall between maternal IDP and offspring ADHD [16, 17]. It should be noted that among those studies, Dreier et al. [16] on additional analysis, did report an association with fever in early gestation as well as genitourinary infection in late gestation and ADHD. These findings are in contrast with a previous twin study [18] and other clinical [12, 13] as well as population-based studies [14, 15] reporting an association with various maternal infections in pregnancy and ADHD in children. However, these findings differ from studies showing association between maternal diseases with immune components and ADHD [4–6].

Several studies using animal models have demonstrated prenatal infection and subsequent maternal immune activation affecting fetal brain development and producing changes in both gray matter and white matter, similar to that seen among patients with neuropsychiatric disorders [40, 41]. Birth cohort studies using maternal biomarkers of infection and inflammation obtained from pregnant maternal serum have demonstrated associations between elevated maternal CRP and schizophrenia [7] and ASD [10]. However, the lack of association seen with similar studies on BPD [42] suggests existence of different pathways linking maternal immune activation and development of various neuropsychiatric disorders. The fact that we used nearly identical study designs in three prior studies and the present study, all of which are based on the same national birth cohort, with large sample sizes and strong statistical power, supports the specificity of maternal CRP as a risk factor for schizophrenia and autism, and not for ADHD and bipolar disorder. With regard to other studies, differences in the study design, methods for ascertainment of maternal infection exposure, as well as the timing of biomarker obtained, either during pregnancy or after childbirth limits straightforward comparisons. It should be noted that we have not ruled out an effect of maternal infection on ADHD but rather the study strongly suggests that general peripheral maternal inflammation at least during early gestation is not related to this outcome. It will be important, therefore, to not only replicate the negative association with CRP but also evaluate infectious and other inflammatory exposures at later stages of pregnancy and ADHD in future work.

We also comment on results of a few additional analyses. A stratified analysis of ADHD cases separately for males and females was used to evaluate any role of offspring sex in the association. To assess the possible contribution of comorbid disorders on the association between maternal CRP and offspring ADHD, we stratified the cases into lifetime comorbid ASD and CD. There were no significant associations seen in both sex and comorbidity stratified analyses.

ASD is also known to be comorbid with ADHD. The findings of this study argue that our previous finding of an association between maternal CRP and ASD [10] is likely not accounted for by this comorbidity.

The strengths of the study include: (1) availability of CRP levels from prospectively drawn maternal serum samples collected during early to mid-gestation that allows a greater focus on prenatal influences. (2) ADHD cases and controls identified from nationwide registers providing a representative study sample, (3) availability of information on several potential confounders including parental psychopathology that was missing in several earlier studies, (4) this is the largest study to date using a biomarker of prenatal inflammation in relation to offspring ADHD.

The study does have a few limitations that need to be considered. First, the ADHD cases included only those that were referred to specialized services, and thus likely represent more severe cases. However, a previous study has reported an 88% validity of the ADHD diagnoses in the FHDR examined against DMS-IV criteria for ADHD. Second, the study examined a single marker of infection/inflammation with ADHD. The availability of several inflammatory markers would have possibly enabled examination of more specific markers of inflammation but that was not possible due to the predefined study protocol. Third, the study had maternal CRP measurement at only one time point in early pregnancy and thus the lack of findings seen may not be applicable to infections and inflammation occurring later during pregnancy. Fourth, despite having a wide array of confounders, the study lacks information on an important confounder, maternal body mass index (BMI). In addition, despite the availability of information on parental ADHD diagnosis, the number of parents with ADHD diagnoses in the sample was low, which is a study limitation. The possible reason for underdiagnoses among parents could be the fact that ADHD was not a widely used diagnosis in the parental generation. Furthermore, the FHDR does not cover outpatient diagnoses before 1998, and thus the diagnosis of ADHD among parents is likely underestimated as most of them are likely to have been treated in outpatient care. Lastly, although biodegradation is a theoretical possibility, this is very unlikely. Over the years, CRP analyses have been run from thousands of samples stored in the Finnish Maternity Cohort (FMC) with varying time differences between sampling and laboratory analysis. Our analysis of CRP levels between samples analyzed 10 years after sampling and those analyzed between 15 and 30 years after sampling showed no variation in the CRP levels (unpublished data). In addition, since the sampling from cases and controls was drawn during the same time period and thus stored for equal lengths of time, the case–control design effectively eliminates out any potential effects of storage.

## Conclusion

This study examined for the first time the association between maternal CRP and offspring ADHD. The lack of any association, in contrast to significant associations of maternal CRP with ASD and schizophrenia suggests different pathways linking maternal immune activation and development of various neuropsychiatric disorders.
